# The Fungus Among Us: Latency, Reactivation, and the Expanding Clinical Spectrum of Coccidioidomycosis

**DOI:** 10.7759/cureus.107243

**Published:** 2026-04-17

**Authors:** Sumeet Bhardwaj, Arushi Tewari, Rishima Tewari, Simona Kogan

**Affiliations:** 1 Psychiatry, Kansas City University, Kansas City, USA; 2 Biology, University of California, Santa Barbara, Santa Barbara, USA; 3 Dermatology, Philadelphia College of Osteopathic Medicine, Philadephia, USA

**Keywords:** coccidioides immitis, coccidioides posadasii, coccidioidomycosis, disseminated disease, fungal infection, immunocompromised host, reactivation, valley fever

## Abstract

Coccidioidomycosis is a serious fungal infection that can produce a wide range of clinical manifestations, from mild, self-limiting pulmonary disease to severe, disseminated infection that can involve nearly any organ system, including the skin. Coccidioidomycosis may also affect patients at the psychiatric level. Its clinical relevance has increased markedly in recent years, as incidence continues to climb, geographic distribution widens, and emerging presentations challenge traditional assumptions regarding fungal latency and host immunity. A notably important and developing area of study is the concept of late reactivation. A narrative review of the literature was conducted using PubMed, MEDLINE, and Google Scholar. Search terms included coccidioidomycosis, *Coccidioides*, latency, reactivation, disseminated disease, serology, climate change, and fungal epidemiology, among others. Priority was given to peer-reviewed reviews, clinical guidelines, epidemiologic studies, and case series.

Growing evidence indicates that *Coccidioides* can persist in a latent state for many years or even decades after apparent clinical recovery, with the potential to reactivate under conditions of declining host immunity or other physiological stressors. Diagnostic tools are currently inadequate to reliably differentiate latent infection from complete eradication, and no validated biomarkers are available to predict the risk of reactivation. Neuropsychiatric and dermatologic manifestations are underrecognized yet clinically important aspects of the disease burden. Coccidioidomycosis remains a complex and difficult condition to manage. This literature review seeks to highlight existing gaps in the literature and to promote further research on this disease.

## Introduction and background

*Coccidioides immitis (C. immitis)* and *Coccidioides posadasii (C. posadasii)* are dimorphic fungi that reside in the arid and semiarid soils of the Americas. Infection occurs via inhalation of aerosolized arthroconidia, which convert into spherules after deposition in the distal airways. While approximately 60% of infections remain asymptomatic or subclinical, the pathogen is capable of producing a broad clinical spectrum, ranging from self-limited pulmonary illness to severe disseminated disease. Coccidioidomycosis is increasingly recognized not only as a cause of acute pulmonary infection but also as a chronic disease capable of delayed relapse, dissemination, and long-term morbidity [1-2]. Unlike many fungal infections that resolve definitively after treatment or immune recovery, *Coccidioides* spp. possess the capacity to persist silently for years to decades after apparent clinical resolution, with reactivation occurring when host immunity is compromised [1-2]. This phenomenon fundamentally challenges traditional assumptions regarding fungal clearance and underscores major gaps in understanding the mechanisms that govern persistence, immune containment, and relapse [1-3].

Current evidence suggests that *Coccidioides* can evade immune eradication by persisting within granulomatous lesions or intracellular compartments, where fungal elements are shielded from immune surveillance [4-5]. Durable containment appears to depend on sustained Th1-mediated cellular immunity, whereas shifts toward Th2 responses or dysregulated Th17 pathways are associated with chronic or reactivated disease [3,6-8]. However, these immunologic models remain largely inferential, and the lack of reliable markers of persistent fungal viability constrains their clinical applicability. A major challenge in clinical practice is the lack of validated biomarkers or predictive tools to identify individuals at risk of reactivation, particularly among those with remote exposure or prior infection [9].

Serologic assays, including enzyme immunoassay, immunodiffusion, and complement fixation, may revert to negative following clinical recovery, yet seronegativity does not reliably indicate a sterilizing cure, particularly in immunocompromised individuals [10-11]. Similarly, radiographic stability provides limited reassurance, as residual lesions may remain unchanged for years while viable organisms continue to persist [10-11]. As a result, clinicians must often make long-term management decisions based on indirect proxies rather than direct measures of fungal clearance.

Clinicians practicing outside endemic regions may be less likely to recognize histories of remote exposure, allowing delayed reactivation to present with atypical or extrapulmonary manifestations [1,11]. This diagnostic uncertainty often leads to the use of prolonged or indefinite antifungal therapy to reduce the risk of relapse, despite concerns regarding cumulative drug toxicity, financial burden, and patient adherence [10-12]. However, discontinuing therapy based solely on negative serology or stable imaging carries a risk of reactivation, particularly within the central nervous system (CNS), where relapse may be fatal if not promptly identified [10-11,13]. At the population level, the inability to reliably detect latent infection limits accurate estimation of disease burden and complicates the allocation of preventive resources, especially as the endemic range of *Coccidioides* continues to expand due to climate change and increased population mobility [14-16].

The incidence of coccidioidomycosis has risen sharply over the past two decades, driven by climate change, urban development in arid regions, and population movement [15]. Warming temperatures, prolonged droughts, and alternating wet-dry cycles increasingly favor fungal proliferation and aerosolization, with projections suggesting continued expansion beyond traditional endemic zones [17]. The resulting growth in the population with prior infection, many of whom were asymptomatic, raises concern for a large and largely invisible reservoir of latent disease. Host immune responses often contain but do not fully eradicate Coccidioides, allowing long-term persistence after initial exposure [18].

Epidemiology

The Centers for Disease Control and Prevention (CDC) surveillance data indicate approximately 20,000 reported cases of coccidioidomycosis in 2019; however, modeling studies suggest true symptomatic infections may be 10-18 times higher, corresponding to 200,000-360,000 cases annually [15]. Underreporting reflects nonspecific clinical presentations, variable testing practices, and limited clinician awareness outside endemic regions [15]. Hospitalization estimates range from 18,000 to 28,000 annually, with 700-1,100 deaths attributed to the disease [15].

Although Arizona and California account for the majority of reported cases, the endemic zone is expanding. Climate models and surveillance data demonstrate northward and eastward spread into Nevada, Utah, New Mexico, Texas, and Washington state [19]. Incidence in Arizona peaked at 260.5 per 100,000 in 2011 before stabilizing, while California’s incidence increased steadily through 2017, with the highest rates reported in Kern, Tulare, Kings, and Fresno Counties [20-21]. These trends suggest not only increased detection but also genuine expansion of exposure risk, with important implications for latent infection burden. Collectively, these trends highlight coccidioidomycosis as an expanding and often underrecognized public health concern.

## Review

Methods

This narrative review was conducted to synthesize current knowledge on coccidioidomycosis, with a focus on fungal latency, delayed reactivation, diagnostic limitations, and environmental amplification. Latency is operationalized within this review through three distinct but overlapping phenomena. The first is microbiologic dormancy, defined as the persistence of viable but non-replicating fungal elements within host tissue. This state is maintained by immune containment, which refers to the active suppression of fungal proliferation by host cellular immunity without achieving sterilizing eradication, whereas clinical quiescence represents the absence of symptoms despite the presence of a persistent infection.

A comprehensive literature search was performed using PubMed/MEDLINE, Google Scholar, and reference lists of key publications. The final search encompassed literature from database inception to the present. The following reproducible PubMed search string was employed, to ensure methodological rigor: ("coccidioidomycosis"[MeSH Terms] OR "Coccidioides"[tiab]) AND ("latency"[tiab] OR "reactivation"[tiab] OR "relapse"[tiab] OR "disseminated disease"[tiab] OR "serology"[tiab] OR "complement fixation"[tiab] OR "immunodiffusion"[tiab] OR "meningitis"[tiab] OR "central nervous system"[tiab] OR "granuloma"[tiab] OR "immune containment"[tiab] OR "climate change"[tiab] OR "dust storms"[tiab] OR "wildfires"[tiab] OR "ecology"[tiab] OR "rodent reservoir"[tiab] OR "fungal epidemiology"[tiab])[Office2].

Human studies were prioritized, though relevant animal, ecological, and environmental literature was included where necessary to contextualize persistence and transmission dynamics. Inclusion criteria encompassed peer-reviewed original research and reviews, clinical guidelines, longitudinal surveillance data, mechanistic immunology studies, and well-documented case series. Seminal historical publications, such as the foundational work by Smith et al. (1946) [22], were included when essential to disease ecology or pathogenesis. Studies were excluded if they were non-peer-reviewed, lacked sufficient methodological detail, or did not directly pertain to *Coccidioides* species.

The initial search identified 979 publications. Following title, abstract, and full-text screening, a final set of 51 sources was selected for inclusion based on their relevance to the core themes of fungal persistence and reactivation. Given the narrative design, no formal study quality scoring or meta-analysis was performed. This review did not involve human subjects research or require institutional review board approval.

Etiology and microbiology

Coccidioidomycosis is caused by the dimorphic fungi *C. immitis* and *C. posadasii* of the *Onygenaceae* family [1]. In the environment, these organisms exist as mycelial molds within arid and semiarid soil, fragmenting into infectious arthroconidia that become aerosolized when soil is disturbed [1]. Inhalation of arthroconidia leads to deposition in the distal airways, where they undergo conversion into spherules, the parasitic tissue form [18].

Spherules mature within host tissue and release numerous endospores upon rupture, propagating local infection and, in some cases, facilitating hematogenous dissemination [18]. The majority of primary infections are entirely asymptomatic, with disseminated disease occurring in a minority of exposed individuals, particularly those with underlying immunosuppression or specific demographic risk factors [1]. The spherule form is central to virulence, conferring resistance to phagocytosis and modulating host immune responses. Importantly, *Coccidioides* is not transmitted person to person or via animals [1]. While morphologically indistinguishable, *C. immitis* predominates in California’s San Joaquin Valley, whereas *C. posadasii* is more prevalent in Arizona, Texas, Mexico, and Central and South America [1]. Genomic studies demonstrate considerable diversity and expansions in gene families related to environmental survival and host adaptation [1]. Both species are classified as biosafety level-3 pathogens due to high infectivity and laboratory transmission risk [1].

Clinical manifestations

Approximately 60% of *Coccidioides* infections are asymptomatic or subclinical [11,22]. Symptomatic pulmonary disease typically develops after a one-to-three-week incubation period and presents as a self-limited influenza-like illness characterized by fever, cough, pleuritic chest pain, malaise, night sweats, and arthralgias [11]. Cutaneous manifestations such as erythema nodosum or erythema multiforme may occur and are associated with robust cell-mediated immunity [11,22]. Nearly every organ system and soft tissue has been described as infected with *Coccidioides* species [23]. Fatigue and weight loss may persist for weeks or months following resolution of acute symptoms [11,24].

Progression to chronic pulmonary or disseminated disease occurs in approximately 5-10% of cases [11,24]. Chronic pulmonary disease may present months to years later with cavitary lesions, nodules, or fibrosis that mimic tuberculosis or malignancy [11,25]. Disseminated disease results from hematogenous or lymphatic spread and may involve skin, bone, joints, and the central nervous system [11,24,26-28]. Coccidioidal meningitis is uniformly fatal without treatment and requires lifelong antifungal therapy [11,28]. In rare cases, dissemination involves the peritoneum, liver, adrenal glands, lymph nodes, kidneys, or heart [11,24,29-30]. Immune-mediated manifestations such as polyarthritis (“desert rheumatism”) and vasculitic eruptions are also reported [11,31]. A chronic-fatigue-like illness occurs in some individuals [32]. Risk factors for dissemination include immunodeficiency, pregnancy, diabetes, advanced age, and chronic kidney disease [11,33-35].

Relapse risk assessment and rationale for lifelong suppressive therapy

Relapse following antifungal discontinuation remains a major clinical challenge. Recurrence rates for coccidioidal meningitis exceed 80% when azole therapy is stopped, and relapse occurs in 25-33% of non-meningeal disseminated disease after cessation [13]. These data indicate that clinical improvement frequently reflects suppression rather than eradication [13]. Accordingly, lifelong suppressive azole therapy is recommended for coccidioidal meningitis and strongly advised for immunocompromised individuals [11,13]. Current guidelines emphasize indefinite therapy in these populations due to persistent relapse risk despite apparent stability [11,13].

Atypical and extrapulmonary presentations

There are atypical manifestations that commonly lack classic pulmonary symptoms, contributing to delayed diagnosis, particularly in non-endemic regions where clinical suspicion is low [36]. Long-term vigilance is therefore required in individuals with remote exposure histories [36].

Diagnostic challenges

Diagnosis of late or atypical relapse is limited by imperfect diagnostic tools [37]. Complement fixation titers may be low or absent in immunocompromised individuals, rendering serology unreliable as a sole diagnostic modality [37]. Definitive diagnosis often requires tissue sampling with identification of spherules, particularly in bone or soft-tissue disease [11]. Serial serologic testing combined with imaging remains central to monitoring [37]. Adjunctive assays such as antigen detection and polymerase chain reaction (PCR) may improve sensitivity, though availability and performance vary [38]. In practice, clinicians are often forced to manage uncertainty rather than resolve it, relying on prolonged therapy in high-risk cases.

Psychiatric and neuropsychiatric manifestations

Psychiatric and neuropsychiatric manifestations of coccidioidomycosis represent an underrecognized but clinically important dimension of disease burden, particularly in the context of delayed reactivation and chronic infection. These manifestations span emotional, cognitive, and behavioral domains and are most commonly observed in patients with CNS involvement or prolonged systemic illness.

Dissemination of *Coccidioides* to the meninges resulting in coccidioidal meningitis represents one of the most severe expressions of failed immune containment and is frequently accompanied by neuropsychiatric morbidity [37]. Coccidioidal meningitis is almost universally fatal without treatment [37,38], and even among survivors, long-term sequelae such as chronic headache, cognitive impairment, and depression are common [39]. In a retrospective case series from a neurological referral center in Mexico, neuropsychiatric symptoms were reported in 64% of patients with coccidioidal meningitis, with neuroimaging demonstrating cerebral and cerebellar volume loss correlated with cognitive decline and depressive symptoms during follow-up [40]. These findings highlight that CNS disease often produces persistent neuropsychiatric morbidity even when anti-fungal therapy prevents immediate mortality [40].

Importantly, neuropsychiatric manifestations may precede overt meningeal symptoms, further complicating diagnosis. Clinical reports describe insidious presentations in which coccidioidal meningitis initially manifests as progressive cognitive dysfunction, mimicking primary neurodegenerative or psychiatric disorders and delaying appropriate recognition [41]. Such cases underscore how latent infection and atypical reactivation can obscure the infectious etiology, particularly in non-endemic regions or in patients without recent exposure.

Beyond direct CNS involvement, systemic coccidioidomycosis is associated with prolonged fatigue, impaired physical functioning, and reduced quality of life, all of which contribute to psychological distress. Observational studies using validated instruments such as the Fatigue Severity Scale (FSS) and the Short Form-36 (SF-36) demonstrate significantly elevated fatigue in patients with pulmonary coccidioidomycosis compared with healthy controls and reference populations with other chronic diseases [42]. Although gradual improvement may occur, fatigue often persists for months and substantially limits daily functioning. Earlier studies similarly reported severe fatigue in a majority of patients with active disease, with severity correlating with BMI [43].

Claims-based analyses further confirm that symptoms such as chronic fatigue, headache, and weakness frequently persist for 9-12 months following diagnosis, accompanied by elevated rates of anxiety and depression for up to one year [43]. Collectively, these findings indicate that psychiatric morbidity in coccidioidomycosis reflects not only acute disease severity but also the prolonged physiological and psychological consequences of persistent infection, delayed recovery, and uncertainty surrounding true fungal clearance.

Dermatologic manifestations

Cutaneous manifestations of coccidioidomycosis provide visible indicators of host immune response and disease progression and can serve as early or delayed markers of containment failure, dissemination, or reactivation. These manifestations broadly fall into two categories: immune-mediated reactive eruptions and direct fungal involvement of the skin [44]. Reactive cutaneous manifestations occur early in the disease and reflect robust cell-mediated immunity rather than direct fungal invasion. Erythema nodosum, the most common of these reactions, typically develops within weeks of pulmonary symptom onset and is associated with favorable immune containment and lower risk of dissemination [44]. Other reactive eruptions, including erythema multiforme and toxic erythema, similarly represent hypersensitivity responses to systemic infection rather than cutaneous fungal burden [44]. While clinically important, these reactions generally resolve spontaneously and do not indicate persistent or disseminated infection.

In contrast, direct cutaneous involvement by *Coccidioides* signals hematogenous spread or failed immune control and is most often observed in immunocompromised patients or those with severe disease [44]. Primary cutaneous coccidioidomycosis, resulting from direct inoculation, is rare and typically localized; however, secondary cutaneous dissemination reflects systemic spread and frequently accompanies involvement of other organ systems [44,45]. Lesions may develop weeks to months after primary pulmonary infection and often serve as external markers of deeper, ongoing infection.

From a diagnostic perspective, cutaneous disease can provide critical clues to the underlying disease stage. Isolated IgM positivity is more commonly associated with localized primary cutaneous infection, whereas disseminated cutaneous disease is typically accompanied by IgG antibodies detected by complement fixation or immunodiffusion assays, reflecting advanced systemic involvement [44,45]. Importantly, secondary cutaneous dissemination often necessitates prolonged systemic antifungal therapy due to the high risk of relapse and multisystem disease [44,45].

Within the broader framework of coccidioidomycosis, dermatologic manifestations illustrate the spectrum of host-pathogen interaction from effective immune containment to overt dissemination and reinforce the central challenge of distinguishing resolved infection from persistent disease. As with neuropsychiatric sequelae, cutaneous findings frequently emerge after initial pulmonary illness, emphasizing how delayed manifestations can reveal latent infection long after apparent clinical resolution.

Ecological connections

**Figure 1:**
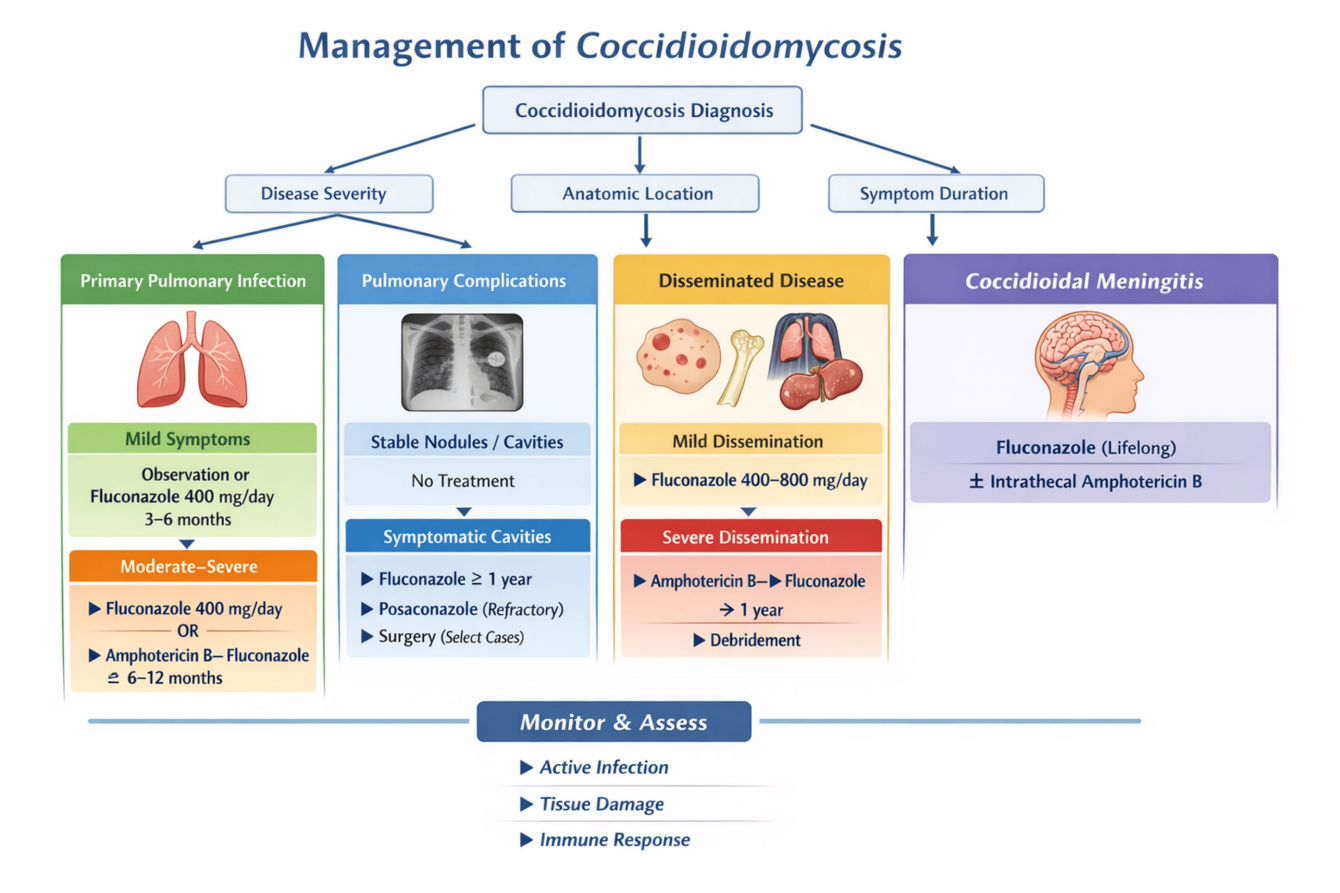
Clinical management algorithm for coccidioidomycosis Decision pathways are stratified by disease severity, anatomic location, and symptom duration. Treatment for primary pulmonary infection ranges from observation to 12 months of antifungal therapy, depending on severity. Management of pulmonary complications distinguishes between stable lesions and symptomatic cavities. Disseminated disease is categorized by severity, with severe cases requiring induction with amphotericin B. Coccidioidal meningitis requires lifelong suppressive therapy. Monitoring encompasses assessment of active infection, tissue damage, and host immune response Image credits: Sumeet Bhardwaj; information derived from established clinical guidelines [11,13]

Coccidioidomycosis involves a complex ecological relationship with rodents and other small mammals [46]. The endozoan reservoir hypothesis suggests that *Coccidioides* may persist within granulomas of small mammals, remaining dormant until host death, after which the fungus re-enters the soil and resumes environmental growth [47]. This interaction likely contributes to localized environmental enrichment and exposure “hot spots,” particularly when combined with habitat disturbance. Tables 1-2 present relevant information on *Coccidioides* and its historic range, including risk factors. Figure 1 provides a visual representation of the potential treatment regimes for coccidiomycosis.

**Table 1:** Coccidioides species that can cause infection and their historic range C. posadasii: Coccidioides posadasii; C. immitis: Coccidioides immitis

Region	Species	Key characteristics
Annual U.S. Statistics	Both *C. posadasii* and *C. immitis*	~150,000 infections annually; ~50,000 symptomatic; 10,000–20,000 diagnosed; 600–1,000 disseminated; ~160 deaths [11]
Arizona (south-central) [11]	*C. posadasii* [11]	Major endemic area; cases increased 10-fold from 1998 to 2011 [11]
Southwestern U.S. (New Mexico, Texas) [11]	*C. posadasii* [11]	Low desert regions; southeastern Washington has unexpected endemic pockets [11]
Mexico, Central and South America [11]	*C. posadasii* [11]	Widely distributed; cases rising in Argentina, Brazil, and Mexico [48]
California (San Joaquin Valley)	*C. immitis* [11]	Associated with the highest incidence of coccidiomycosis in the United States [49]

**Table 2:** Risk factors for Coccidioides infection HIV: human immunodeficiency virus; AIDS: acquired immunodeficiency syndrome; TNF-α: tumor necrosis factor-alpha; IFN-γ: interferon gamma; IL-12: interleukin 12

Risk factor category	Specific factors	Clinical impact
Immunosuppression	HIV/AIDS; primary immunodeficiency [11]	Major risk for dissemination and severe disease [11]
Medications	Corticosteroids, TNF-α inhibitors [11]	Impaired T-cell mediated immunity [50]
Age	>60 years [11]	Vulnerable population for severe disease [11]
Pregnancy	All trimesters of pregnancy [11]	Risk for severe/progressive disease [11]
Comorbidities	Diabetes mellitus [11, 51]	Associated with more severe disease [11,51]
Immune defects	IFN-γ/IL-12 pathway defects [11]	Severe infections in children [11]

Discussion 

This review highlights coccidioidomycosis as a pathogen whose clinical significance extends far beyond acute infection. The capacity of *Coccidioides* spp. to persist in a dormant state for years to decades challenges traditional assumptions of fungal clearance and underscores major gaps in our understanding of host-pathogen equilibrium [10]. The immunologic mechanisms governing latency and reactivation remain incompletely defined. While murine models have provided mechanistic proof that Th17 and Th1 pathways are essential for fungal control, evidence in humans is primarily restricted to clinical correlations. In human cohorts, durable containment appears to depend heavily on sustained Th1-mediated cellular immunity, whereas disseminated or relapsed disease is often associated with diminished interferon-gamma production and a shift toward Th2-skewed responses [3,7]. However, the role of Th17 in human latency remains largely inferential and lacks the definitive mechanistic confirmation seen in animal studies. The inability to directly detect viable dormant organisms in vivo further complicates efforts to define latency, leaving clinicians without reliable biomarkers to assess eradication versus persistence [13].

Current diagnostic tools, including serologic assays and radiographic monitoring, are insufficient to resolve this uncertainty, as they often revert to negative or remain stable despite the presence of viable organisms [10]. Emerging molecular and antigen-based assays are under investigation to address this biomarker gap. Quantitative PCR (qPCR) and metagenomic next-generation sequencing (mNGS) have shown promise in detecting low-level fungal DNA in clinical samples, potentially offering higher sensitivity than traditional culture. Furthermore, the development of specialized *Coccidioides* antigen detection assays and T-cell interferon-gamma release assays (IGRAs) may eventually provide objective measures of both fungal burden and host T-cell vigor, respectively.

This diagnostic uncertainty has profound clinical consequences, particularly in high-risk populations. The inability to definitively confirm sterilizing cure forces clinicians to rely on imperfect clinical proxies when making high-stakes decisions, such as discontinuing antifungal therapy or initiating prophylaxis [51,13]. The high relapse rates observed after antifungal discontinuation, particularly in *Coccidioides* meningitis, reinforce the concept that, while most infections remain asymptomatic or self-limited, *Coccidioides* behaves more like a chronic infection than a self-limited mycosis in a clinically important subset of patients [9,13]. This reality explains the widespread reliance on lifelong suppressive therapy in selected populations, despite concerns regarding toxicity, cost, and long-term adherence.

Future research directions

To handle the expected surge in cases of coccidioidomycosis, future research should focus on prioritizing direct or near-direct detection of persistent infection, alongside mechanistic studies clarifying the immunologic events that precede reactivation in individuals who previously had the disease. Clinical studies should test evidence-based strategies for prophylaxis in patients with remote infection who later undergo immunosuppression, with endpoints that include relapse, dissemination, toxicity, adherence, and quality of life. In addition, because coccidioidomycosis also imposes substantial neuropsychiatric and functional morbidity, future longitudinal studies should incorporate validated fatigue, cognitive, and mental health instruments and evaluate whether earlier diagnosis, optimized antifungal regimens, and rehabilitative or psychiatric interventions improve long-term outcomes. Lastly, improved environmental surveillance and modeling are essential to anticipate new endemic zones and quantify the growing latent reservoir created by expanding exposure.

## Conclusions

Coccidioidomycosis represents an underrecognized challenge at the intersection of infectious disease, immunology, and environmental change. Shifting environmental conditions and increasing population mobility are likely expanding the pool of individuals with remote or unrecognized exposure, raising concern that delayed reactivation and diagnostic uncertainty will become increasingly common. Addressing this hidden burden will require improved understanding of fungal latency, better strategies for risk assessment, and heightened clinical awareness, especially outside traditionally endemic regions. Without such advances, latent infection will continue to evade detection until reactivation occurs, often with significant clinical consequences.
